# Cholera Rapid Test with Enrichment Step Has Diagnostic Performance Equivalent to Culture

**DOI:** 10.1371/journal.pone.0168257

**Published:** 2016-12-19

**Authors:** Lameck N. Ontweka, Lul O. Deng, Jean Rauzier, Amanda K. Debes, Fisseha Tadesse, Lucy A. Parker, Joseph F. Wamala, Bior K. Bior, Michael Lasuba, Abiem Bona But, Francesco Grandesso, Christine Jamet, Sandra Cohuet, Iza Ciglenecki, Micaela Serafini, David A. Sack, Marie-Laure Quilici, Andrew S. Azman, Francisco J. Luquero, Anne-Laure Page

**Affiliations:** 1 Médecins Sans Frontières Operational Center Geneva, Geneva, Switzerland; 2 Amref Health Africa Headquarters, Nairobi, Kenya; 3 National Public Health Laboratory, Ministry of Health, Juba, South Sudan; 4 Enteric Bacterial Pathogens Unit, National Reference Centre for Vibrios and Cholera, Institut Pasteur, Paris, France; 5 Department of International Health, Johns Hopkins University, Baltimore, United States of America; 6 Field epidemiology, Epicentre, Paris, France; 7 World Health Organization, Juba, South Sudan; 8 Department of Epidemiology, Johns Hopkins University, Baltimore, United States of America; Wadsworth Center, UNITED STATES

## Abstract

Cholera rapid diagnostic tests (RDT) could play a central role in outbreak detection and surveillance in low-resource settings, but their modest performance has hindered their broad adoption. The addition of an enrichment step may improve test specificity. We describe the results of a prospective diagnostic evaluation of the Crystal VC RDT (Span Diagnostics, India) with enrichment step and of culture, each compared to polymerase chain reaction (PCR), during a cholera outbreak in South Sudan. RDTs were performed on alkaline peptone water inoculated with stool and incubated for 4–6 hours at ambient temperature. Cholera culture was performed from wet filter paper inoculated with stool. Molecular detection of *Vibrio cholerae* O1 by PCR was done from dry Whatman 903 filter papers inoculated with stool, and from wet filter paper supernatant. In August and September 2015, 101 consecutive suspected cholera cases were enrolled, of which 36 were confirmed by PCR. The enriched RDT had 86.1% (95% CI: 70.5–95.3) sensitivity and 100% (95% CI: 94.4–100) specificity compared to PCR as the reference standard. The sensitivity of culture versus PCR was 83.3% (95% CI: 67.2–93.6) for culture performed on site and 72.2% (95% CI: 54.8–85.8) at the international reference laboratory, where samples were tested after an average delay of two months after sample collection, and specificity was 98.5% (95% CI: 91.7–100) and 100% (95% CI: 94.5–100), respectively. The RDT with enrichment showed performance comparable to that of culture and could be a sustainable alternative to culture confirmation where laboratory capacity is limited.

## Introduction

Cholera continues to be a major public health problem for developing countries with an estimated 2.8 million cholera cases and around 100,000 deaths each year worldwide [1]. Countries with the highest incidence rates are in Africa, Southern Asia and the Caribbean, where surveillance systems are often insensitive and unable to rapidly detect the transmission of epidemic pathogens [2].

Rapid identification and confirmation of initial cases in the early phase of cholera epidemics is critical for timely public health responses to control outbreaks. Diagnostic delays may result in higher case numbers and case fatality rates, leading to an enormous health and economic burden to affected countries. Currently, isolation of *Vibrio cholerae* O1 by stool culture is necessary for cholera outbreak confirmation and remains the gold standard for diagnosis [2]. However, this procedure requires laboratory infrastructure, adequate transport procedures and well trained staff. Moreover, the delay in obtaining results includes the 2 to 3-day duration of the microbiological procedure, in addition to the time for transportation of the sample to the closest laboratory. Culture sensitivity is also imperfect and can be affected by the delays in transport to the laboratory, as well as prior consumption of antibiotics [3]. Polymerase chain reaction (PCR) is becoming more commonly used to detect *V*. *cholerae*, with the advantages of being faster and generally more sensitive than culture [3–5]. However, like culture, PCR requires laboratory infrastructure and well trained staff, which are often not available at sites where cholera outbreaks occur.

A rapid diagnostic test (RDT) that is accurate, simple, easy to use and interpret, and stored at ambient temperature would be a useful tool for the early detection and confirmation of cholera outbreaks. Several rapid tests for cholera have been developed, based mostly on lateral flow immunochromatographic techniques [6]. One of the most widely used RDTs for cholera, Crystal VC^TM^ (Span Diagnostics, Surat, India) or its prototype developed by Institut Pasteur (IP), have been studied in different contexts, and have consistently shown high sensitivities (92–97%), but moderate specificities (49–80%) when used directly on bulk stools and compared to culture as the gold standard [6–11]. While the moderate specificity may be partly explained by the imperfect nature of culture as the gold-standard, analyses including PCR or using statistical models taking into account this bias only increased the specificity to 85% [4].

Alkaline peptone water (APW) is a commonly used enrichment medium for *V*. *cholerae*. In initial assessments of the prototype developed by IP, the test was also evaluated on rectal swabs inoculated in APW and incubated at room temperature for 4 hours [7,12]. This latter method gave a much higher specificity, up to 97%, compared to using the RDT directly on bulk stool. Both methods are now included in the package insert of Crystal VC: the “initial screening” method on direct stool diluted in a sample processing reagent, and the “confirmation” method on stool incubated in APW for 4 hours. So far, only one study conducted in Bangladesh has evaluated the performance of both methods with the current version of the Crystal VC test, and showed that the enriched “confirmation” method had both higher sensitivity and higher specificity compared to the direct screening method [13]. However, in this study, the performance of the direct test was not consistent with previously published studies, with low sensitivity (65%) and high specificity (92%); the reasons for these discrepancies were not discussed, but could be due to the addition of a step of dilution of the stools in a sample preparation vial. Another study confirmed the high specificity of RDT after enrichment in APW but sensitivity could not be reliably estimated due to the low number of confirmed cholera cases [14].

In this study we evaluated the performance of Crystal VC used after a 4–6 hour enrichment in APW compared to PCR as the gold standard method during a cholera outbreak in Juba, South Sudan.

## Materials and Methods

### Ethics

Ethical approval for this study was obtained from the John Hopkins Bloomberg School of Public Health Institutional Review Board and the South Sudan Ministry of Health, Directorate of Monitoring, Evaluation and Research. Written informed consent was obtained from all participants enrolled in the study with parental or guardian consent for participants under 18 years of age.

### Study population

This study was conducted in Juba, the capital of South Sudan, during a cholera outbreak that lasted for five months from May to September 2015. This study was nested in a vaccine effectiveness study following a large oral cholera vaccination campaign [15]. The study population consisted of all individuals aged one year and older presenting with three or more loose stools in the preceding 24 hours at participating cholera treatment centres (CTCs) and oral rehydration posts around the city in August and September 2015.

Written informed consent to participate in the study was obtained once the patient was in stable condition. If no consent was provided, the stool sample was tested for local surveillance purposes, but the results were not used in the study.

We subsequently excluded all patients who reported to have taken the vaccine within 7 days prior to sample collection, since rapid test can become positive in vaccine recipients for up to 6 days after vaccination [16].

### Sample collection and processing

Upon admission, a stool sample was collected in a clean (non-disinfected), unused container. Two drops of watery stool were transferred to a vial containing 3 ml of APW broth and kept at ambient temperature for 4 to 6 hours.

Study staff soaked two 6-mm filter paper disks in each fresh watery stool sample and then placed each filter paper into separate microtubes with two drops of normal saline. One microtube was sent immediately to the National Public Health Laboratory (NPHL) in Juba, and the other was stored at ambient temperature until the end of the study and sent to Institut Pasteur (IP), Paris, France.

Using a Pasteur pipette, one to two drops of direct stool or enriched APW medium were spotted onto a Whatman 903 Protein Saver Card (GE Healthcare Ltd., Forest Farm, Cardiff, UK) and allowed to air-dry. Dry filter papers were packed individually with a desiccant bag and stored at ambient temperature until processing. Dried filter papers were sent to Johns Hopkins University (JHU) to be tested for *V*. *cholerae* using molecular methods.

### Rapid test procedure

Rapid tests were performed at the CTC by three nurses, who were trained on the study procedures (including rapid tests) for two days prior to the study start. RDT kits were stored at ambient temperature.

For the enriched method, after the 4–6 hour incubation of APW at ambient temperature, two drops of enriched medium were placed in the test tube and the dipstick was inserted. The result was read after 15 minutes by trained study staff, and interpreted following the manufacturer’s recommendation. The test was considered positive if the control line and either line T2 (O1) or T1 (O139) or both (O1 and O139) showed pinkish red lines, negative if the control line only showed a pinkish red line and invalid if the control line did not show any coloration. The staff reading the enriched test were not blinded to the results of the direct test, but were blinded to the results of culture and PCR. A picture of each test was taken and results were re-confirmed by the study co-investigators.

Rapid tests were also performed using two drops of direct stool. Since this procedure did not strictly follow the manufacturer’s recommendations, which includes dilution in a sample diluent buffer, we did not include the results in the main analysis and provide the corresponding data in S1 Appendix.

### Stool culture

Upon arrival in both laboratories, culture was performed from the wet filter papers by trained laboratory technicians using standard methods including enrichment in APW [17]. Briefly, a loopful of supernatant from the wet filter papers was cultured on thiosulfate-citrate-bile salt-sucrose (TCBS) agar and, at NPHL, on MacConkey agar, as selective plating media, and on blood agar or alkaline nutrient agar as nonselective plating media. In addition, the wet filter papers were placed in APW solution and incubated for 6–8 hours at 37° C. After incubation, one loopful of APW from the topmost portion of the broth, where *V*. *cholerae* preferentially grows, was sub-cultured on selective and nonselective media as described above. Culture plates were incubated overnight at a temperature of 35–37°C. Screening of isolates was based on morphological appearance on selective and nonselective media, Gram staining, motility and oxydase testing. Identification was confirmed by serological testing performed using polyvalent O1- and O139-specific antisera (Bio-Rad, USA and Denka Seiken Co, Japan, respectively) and serotype determination by monovalent Inaba and Ogawa antisera (Denka Seiken Co, Japan), all performed on colonies grown on nonselective media.

The median delay between sample collection and culture was 1 day in Juba and 73 days (range: 26 to 82 days) in Paris.

### Polymerase Chain Reaction (PCR) analysis

In the absence of an internationally recognized standard method for molecular detection of *V*. *cholerae* O1, PCR was performed in parallel in two laboratories both on different matrices and different target genes.

At JHU, DNA from each dried filter paper specimen was extracted using chelex-100 (Bio-Rad) and subsequent PCR amplification was performed for the detection of the outer membrane protein of *V*. *cholerae*, *ompW*, for species confirmation; the cholera toxin A gene, *ctxA*, to assess the toxigenic potential of the strains [18]; and the *rfb* gene for the identification of the O1 or O139 serogroups [19]. All negative samples were further tested for the presence of 16S rDNA to confirm DNA preservation techniques as described by Hasan *et al*. [20]. PCR was performed according to methods previously described [14].

At IP, DNA was extracted from the wet paper supernatant and from the enrichment in APW by boiling the samples for 10 minutes at 100°C. PCR was performed to detect an intergenic spacer region specific of *V*. *cholerae* species (ISR gene) [21]. On samples positive for *V*. *cholerae*, the *rfb* gene was amplified for the identification of *V*. *cholerae* serogroup O1 and O139 as described by Hoshino [19]. On negative samples, PCR was performed on a 1/10 dilution of the target DNA to check for the presence of inhibitors.

### Data analysis

The reference standard was considered as positive if the PCR result was positive for *V*. *cholerae* O1 either at JHU or at IP. For the main analysis, the RDT was considered positive if the O1 line was positive and negative if the O1 line was negative, irrespective of the result of the O139 line. Additional analysis was performed considering the RDT as positive if either the O1 or the O139 line was positive, and negative if both lines were negative. Culture was considered positive if *V*. *cholerae* O1 was isolated from stool and negative if no *V*. *cholerae* was found, or if non-O1 non-O139 *V*. *cholerae* was isolated.

Data analysis was performed using Stata 13 (Stata Corporation, College Station, Texas, USA), with the estimates of diagnostic performance produced using the *diagt* command, which displays summary statistics for diagnostic tests and provides exact binomial confidence intervals for sensitivity, specificity, and predictive values.

For the comparison between rapid test and culture performance, we used McNemar’s chi-2 test and applied it globally, as well as separately on samples that were positive and negative by the reference standard to assess for possible statistical differences in sensitivity and specificity. We also used the Cohen’s kappa coefficient to assess global agreement between the assays.

## Results

From August 9 to September 29, 2015, 110 patients attending the study health facilities were screened for inclusion in the study. One patient was excluded because the stool sample was not collected and five did not provide informed consent. In addition, three patients who reported to have received a single dose of oral cholera vaccine within 7 days prior to consultation were excluded. In total, 101 patients were included in these analyses, with a majority of males (n = 59/99, 59.6%) and a median age of 26 years (interquartile range: 8–35). Of 83 patients with clinical information available, most had severe dehydration (n = 51, 61.5%), 11 (13.3%) reported having received antibiotics prior to admission and 16 (19.3%) received antibiotics at the CTC before sample collection. Twelve (12.0%) reported to have taken a single dose of oral cholera vaccine more than one week (range 8–35 days) prior to the consultation.

The RDT with enrichment was positive for O1 in 31, with a weak line in 2 of them (6.5%). None of the enriched RDTs had a positive O139 reading. Culture was positive for *V*. *cholerae* O1 in 31 patients at the Juba NPHL and in 26 at IP (Table 1). PCR was positive for *V*. *cholerae* O1 from 31 wet filter papers at IP and from 35 dry filter papers at the JHU laboratory, resulting in a PCR-positive result in 36 (35.6%) of the 101 specimens.

**Table 1:** Results of the enriched RDT and of culture at National Public Health Laboratory, Juba, and at Institut Pasteur, Paris, compared to PCR results.

		PCR *V*. *cholerae* O1 (reference standard)		
		Positive	Negative	Total
Enriched rapid test				
	Positive O1	31	0	31
	Negative	5	64	69
	Not done	0	1	1
Culture—NPHL				
	Positive	30	1	31
	Negative	6	64	70
Culture—IP				
	Positive	26	0	26
	Negative	10	65	75
Total		36	65	101

When compared to PCR as the reference standard, the enriched test showed moderate sensitivity (86.1%) but very high specificity (100%), which resulted in a 100% positive predictive value (Table 2). While on-site culture showed similar performance to the enriched rapid test (exact McNemar chi-2 test p = 1 globally and for PCR-positive and negative samples separately, kappa = 81.3%), delayed culture at IP showed a lower sensitivity of 72.2% (exact McNemar chi-2 test p = 0.27 globally and for PCR-positive samples, p = 1 for PCR-negative samples, kappa = 68.2%). Excluding patients with self-reported antibiotic intake either before or at the CTC did not change the performance significantly (Table 2).

**Table 2:** Diagnostic performance of direct and enriched RDT, and of culture at National Public Health Laboratory, Juba, and at Institut Pasteur, Paris, using PCR as the reference standard in all (N = 101) or patients without prior antibiotics (N = 80).

		Sensitivity	Specificity	PPV	NPV
		% (95% CI)	% (95% CI)	% (95% CI)	% (95% CI)
All					
	Enriched RDT	86.1 (70.5–95.3)	100 (94.4–100)	100 (88.8–100)	92.8 (83.9–97.6)
	Culture NPHL	83.3 (67.2–93.6)	98.5 (91.7–100)	96.8 (83.3–99.9)	91.4 (82.3–96.8)
	Culture IP	72.2 (54.8–85.8)	100 (94.5–100)	100 (86.8–100)	86.7 (76.8–93.4)
No prior antibiotics					
	Enriched RDT	87.5 (67.6–97.3)	100 (93.6–100)	100 (83.9–100)	94.9 (85.9–98.9)
	Culture NPHL	87.5 (67.6–97.3)	98.2 (90.4–100)	95.5 (77.2–99.9)	94.8 (85.6–98.9)
	Culture IP	70.8 (48.9–87.4)	100 (93.6–100)	100 (80.5–100)	88.9 (78.4–95.4)

## Discussion

This field evaluation of the enriched rapid test for diagnosis of cholera confirmed initial reports suggesting that the APW incubation step ensures high specificity, which improves confidence in the test results and could diminish the risk of false cholera outbreak alerts [13,14]. Incubation in APW for 4–6 hours is also the first step in laboratory methods used for the diagnosis of *V*. *cholerae* from faecal specimens by culture. The subsequent steps of sub-culture and classical bacteriological identification are replaced here by the RDT for detection of *V*. *cholerae* O1 or O139. When used on enriched specimens, the RDT proved that it was sufficiently sensitive and specific for rapid and accurate detection and identification of *V*. *cholerae*, making this method comparable to a simplified and rapid version of culture-based identification of *V*. *cholerae*. This is confirmed by the fact that the test had similar performance as culture when performed within 1 or 2 days of stool collection, as at the Juba NPHL laboratory, and even better performance than culture in case of long delay between sampling and testing, as in the IP laboratory, where culture was performed more than 2 months after sampling.

One possible disadvantage of the enriched method is that, like culture, it depends on the presence of culturable organisms, while the direct test could also detect non-viable and non-culturable organisms. Although it is reasonable to assume that under normal conditions of use of the tests in the field, the absence of viable organisms in the stools of cholera patients is unlikely, it is important to highlight that enrichment could be affected by problems linked to sample collection and storage, such as containers with disinfectants, poor sampling or handling practices with long delays or inappropriate temperature, or to prior antibiotic consumption. Here, exclusion of patients with self-reported previous antibiotic consumption did not significantly change the test performance, but numbers were too limited for a formal stratified analysis, and the class of antibiotics and delay between intake and testing were not known.

There were several limitations to this study. First, the sample size was low, due to the fact that the study was undertaken when the epidemic was already declining and after an OCV vaccination campaign. Second, although the RDT was also performed without enrichment, the fact that we did not strictly follow the current manufacturer’s recommendations and did not use the sample diluent buffer for the direct method prevented us from doing a formal comparison between these methods. The reasons for not strictly following the recommended procedure was due to fear of a decreased sensitivity with the dilution of ~200 μL of stool in 1 mL diluent buffer and to habits with previous versions of the test. Indeed, it should be noted that the initial version of Crystal VC recommended that the test be performed directly on liquid stool, with no diluent, and all but one of the evaluations of Crystal VC published until now used this previous method. Only one study published so far was done with the current version of Crystal VC using the sample diluent buffer and showed the lowest sensitivity reported so far (66%) and high specificity of 92% [13]. Thus, we cannot exclude the possibility that the dilution itself, rather than enrichment in APW, might increase the specificity. However, it seems that dilution alone does not eliminate the problem with false positive O139 results with the direct RDT method, as encountered here (see S1 Appendix), since this issue was also reported after dilution in the sample diluent [13]. In addition, the results from the study in Bangladesh suggests that the use of the sample diluent could reduce the RDT sensitivity to unacceptably low levels.

In conclusion, our results show that the RDT used with a simple step of enrichment in APW has performance similar to that of culture. This method could be a sustainable alternative to culture confirmation of cases in places where laboratory capacity is limited. Culture will remain needed for phenotypic analysis, including antibiotic susceptibility testing, and further molecular characterization, which is essential for worldwide disease surveillance.

## Supporting Information

S1 Appendix Results and performance of the RDT performed directly on stool. (DOCX)

S1 Dataset Study dataset. (XLSX)
