# Supplementary material for: Cholera Rapid Test with Enrichment Step Has Diagnostic Performance Equivalent to Culture
Source: PLoS One. 2016 Dec 19;11(12):e0168257. doi: 10.1371/journal.pone.0168257 (PMC5167375; doi:10.1371/journal.pone.0168257)
Supplement: S1 Appendix — (DOCX) [file pone.0168257.s001.docx]

**Cholera rapid test with enrichment step has diagnostic performance equivalent to culture**

**S1 Appendix**

**Results and performance of the RDT performed directly on stool**

Here, we present the results and performance of the rapid diagnostic test (RDT) Crystal VC performed directly on stools using the same specimens as those described in the main manuscript. The reason for not including the results in the main manuscript is that the direct RDT was not performed using the current manufacturer’s recommendations. It should be noted however, that the method used here corresponds to the recommendations in the initial version of Crystal VC, and that most of the evaluations of this RDT published so far used this method.

Methods – Direct rapid test

For the direct RDT method, two to four drops (~200μl) of stool were placed directly in the RDT test tube and the dipstick was inserted. This was different from the method recommended by the manufacturer, which includes a dilution of two drops of stools with 1 mL of sample diluent included in the kit.

The result was read after 15 minutes by trained study staff, and interpreted following the manufacturer’s recommendation. The test was considered positive if the control line and either line T2 (O1) or T1 (O139) or both (O1 and O139) showed pinkish red lines, negative if the control line only showed a pinkish red line and invalid if the control line did not show any coloration.

Results – Direct rapid test

Of the 101 specimens tested, 18 were positive for O1 only, 29 for O1 and O139 and 7 were positive for O1 only. This resulted in a sensitivity of 94.4% (95% CI: 81.3-99.3) and a specificity of 79.7% (95% CI: 67.8-88.7) when considering only the tests that were positive for O1 (alone or with O139). When considering either O1 or O139 lines as positive, the specificity of the direct rapid test decreased to 68.8% (95% CI: 55.9-79.8).

Supporting Table 1. Results of the direct RDT compared to PCR

|  |  | PCR *V. cholerae* O1 (reference standard) | | |
| --- | --- | --- | --- | --- |
|  |  | Positive | Negative | Total |
| Direct rapid test | |  |  |  |
|  | Positive O1 | 17 | 1 | 18 |
|  | Positive O1 & O139 | 17 | 12 | 29 |
|  | Positive O139 | 0 | 7 | 7 |
|  | Negative | 2 | 44 | 46 |
|  | Invalid | 0 | 1 | 1 |
| Total | | 36 | 65 | 101 |
